# Supplementary material for: Pathophysiological profile of non-ventilated lung injury in healthy female pigs undergoing mechanical ventilation
Source: Commun Med (Lond). 2024 Feb 15;4:18. doi: 10.1038/s43856-024-00449-3 (PMC10869686; doi:10.1038/s43856-024-00449-3)

Pathophysiological profile of non-ventilated lung injury in healthy female pigs undergoing mechanical ventilation

Elena Spinelli, Anna Damia, Francesco Damarco, Beatrice Gregori, Federica Occhipinti, Zara Busani, Marco Leali, Michele Battistin, Caterina Lonati, Zhanqi Zhao, Alessandra Storaci, Gianluca Lopez, Valentina Vaira, Stefano Ferrero, Lorenzo Rosso, Stefano Gatti, Tommaso Mauri

Supplementary Information

Supplementary Methods

The study was approved by the Italian Ministry of Health, Rome, Italy (Aut. No. 246/2022-PR, Protocol No. 568EB.34 (ex 32)) and conducted according to the European Directive 2010/63/EU on the protection of animals used for scientific studies and the Italian decree 26/2014. Approval by the Institutional Animal Care Committee was obtained before starting the experiments.

Animal preparation. In compliance with local recommendations, pigs arrived at the experimental facility the day before the start of the study and fasted overnight with free access to water. Sedation was administered by intramuscular injection of medetomidine 0.025 mg/kg and tiletamine/zolazepam 5 mg/kg. Then, an auricular vein was cannulated and, after administration of cefazoline 1 g and tramadol 50 mg, continuous intravenous (IV) infusion of propofol was titrated to maintain the animal on spontaneous breathing and SpO_2 100% while on additional oxygen via face mask. Surgical tracheostomy was performed in the supine position under additional local anesthesia (lidocaine 2%). After a left sided double-lumen endobronchial tube of 37 Fr (for the NVLI-15, NVLI-7.5 groups) and an endotracheal tube of 7.5mm (for the MV-Control group) was inserted through the tracheostomy and fixed, mechanical ventilation was started and general anesthesia and neuromuscular blockade were maintained by IV propofol 5-10 mg/kg/h, medetomidine 2.5-10.0 μg/kg/h and pancuronium bromide 0.3-0.5 mg/kg/h. Correct positioning of the endobronchial tube was assessed with a single-use fiberscope (Ambu® aScope™ 4 Broncho Slim, Ambu, Netherlands). During the whole experiment, oxygen saturation, heart rate, invasive blood pressure, central venous pressure, pulmonary artery pressure and ventilatory waveforms were monitored. Depth of anesthesia was adjusted to ensure no sign of distress, such as unexplained tachycardia, arterial hypertension and/or horripilation. External warming was provided when core body temperature fell below 37.5°C, as well as external cooling when core body temperature rose above 40°C.

Ringer lactate was administered at 100 ml/h during catheters positioning and reduced to 50 ml/h along the whole study, aiming at a zero fluid balance.

Cefazoline 1 g IV and tramadol 50 mg IV were administered every 12 hours. Low molecular weight heparin 2000 IU was administered subcutaneously once per day.

Instrumentation. Vascular accesses were obtained by surgical exposure. An arterial catheter (Arrow® Seldinger, 18 G 8 cm, Prodimed, Teleflex, Ireland) was inserted in the left common carotid artery. A three-lumen central venous catheter (Arrow®, 7 Fr, Teleflex, Ireland) and a pulmonary artery catheter (Swan Ganz®, 5 Fr, Edwards, USA) were introduced in the left external jugular vein. Positioning of pulmonary artery catheter was guided by visualization of pulmonary artery and wedge pressures. An esophageal balloon catheter (Cooper Surgical, USA) was inserted and inflated with the recommended volume of air. Correct positioning and calibration were confirmed by the standard occlusion test with external compressions.

At the end of instrumentation, in animals from NVLI-15 and NVLI-7.5 groups correct positioning of the endobronchial tube was assessed again and finally left lung was excluded from ventilation. Baseline measurements including body temperature, arterial blood gases and respiratory system and lung compliance were taken to exclude pathological conditions before beginning the experiment.

Study measurements. Pulsossimetry, heart rate, invasive arterial pressure and pulmonary artery pressure were continuously monitored. End-tidal CO_2 was measured through a capnometer (Philips, The Netherlands). A 16 electrodes EIT belt was positioned around the swine's chest, connected to an EIT monitor (PulmoVista® 500, Dräger, Lübeck, Germany) and left in place throughout the study period.

Bronchial suctioning followed by a recruitment maneuver (pressure Controlled mode, 45 cmH_2O, I:E=1:1, respiratory rate 10 bpm, for 1 minute/10 breaths) were performed 20 minutes before each timepoint.

The variables collected were:

- Respiratory mechanics: airway peak pressure (Ppeak), plateau pressure (Pplat) by a 3-second inspiratory pause, mean airway pressure (Pmean), total PEEP (PEEPtot) by a 3-second expiratory pause, V_T, change between inspiratory and expiratory esophageal pressure (ΔPes). From

these, driving pressure (DP) was calculated as Pplat –PEEPtot and driving transpulmonary pressure as DP- ΔPes; static respiratory system compliance (C_{RS}) was calculated as V_T/DP; lung compliance (C_L) as V_T/ΔPL; chest wall elastance (C_{CW}) as V_T/ΔPes

- Hemodynamics: systolic, diastolic and mean arterial pressures (SAP, DAP, MAP); systolic, diastolic and mean pulmonary artery pressures (PAPS, PAPD, PAPM); wedge pressure (WP) at end expiration; cardiac output (CO) via thermodilution technique (Vigilance, Baxter Edwards Critical Care, Edwards E6 Lifesciences, USA); heart rate (HR); central venous pressure (CVP) at end expiration, mixed venous oxygen saturation (SvO_2).
- Gas exchange: arterial pH, $PaCO_2$, PaO_2.
- End-tidal CO_2
- EIT data: EIT data were recorded at 50 Hz and stored for offline analysis. EIT ventilation and perfusion maps were obtained by dedicated software. In NVLI-15 and NVLI-7.5 animals, right ventilated lung mask for ventilation and perfusion corresponded to all ventilated pixels, while left non-ventilated lung perfusion was measured in non-ventilated pixels of the left hemithorax. In MV-Control animals, we split the EIT images into two same size regions of interests on the horizontal axis from halfway left and right, roughly corresponding to the right and left lungs. For ventilation maps we averaged values over 8 consecutive respiratory cycles.
- Bronchoalveolar lavage (BAL): right after T24 measurements a bilateral BAL was performed with 30 ml 0.9% saline solution per side. BAL fluids were then centrifugated and the supernatants were promptly stored at -80 °C. Later, these were assayed by ELISA for concentration of regional inflammatory mediators (IL-6, TNFα and IL-1β) and concentration of inflammatory markers of the alveolar epithelium (Surfactant Protein D (SP-D) and Receptor for Advanced Glycation End-products (RAGE)) and endothelium (Angiopoietin 2 (Ang2)), following the manufacturers' instructions.

Euthanasia, autopsy, histology. At the end of the experiment, animals were euthanized (by intravenous injection of potassium chloride 40 mEq under deep sedation) and underwent autopsy for collection of histological samples. Lungs were promptly excised en bloc via sternotomy and the

correct position of the endobronchial tube was confirmed by direct visualization. For each pig's lungs twelve representative tissue samples (2 from upper, 2 from medial and 2 from inferior lobes for each side) of 1 cm^3 volume were collected: 6 were stored in formaldehyde to undergo histological examination and 6 were used to calculate wet to dry ratio. Two pathologists blinded to study group and to the original location of the sample conducted the examination. Right and left histological score of the lungs was calculated from the six samples per animal stored in formaldehyde (3 for each side). Ten main histological alterations were evaluated: emphysematous change, interstitial congestion, alveolar hemorrhage, alveolar neutrophil infiltration, alveolar macrophage proliferation, alveolar type II pneumocytes proliferation, interstitial lymphocytes proliferation, interstitial thickening, hyaline membrane formation and organization of alveolar exudate. Each alteration was scored from 0 (absent) to 3 (severe) in each sample (ten values from zero to 3 for each sample); then, the histological score of that sample was calculated as the sum of the ten values; finally, the regional histological score of the lungs was calculated as the average value of the scores of the 3 samples for each side (range: 0 to 30).

Samples for the wet to dry calculation were weighed before and after being dried for 24 hours at 50°C in an oven. The average value of the scores of the 3 samples for each side was then calculated.

Supplementary results

Supplementary Figure 1. Right and left lung wet-to-dry weight ratio. Both right (panel A) and left (panel B) lung wet-to-dry weight ratio showed low and comparable values among the three study groups.

Data are expressed as scatter plot with bars and error bars (mean ± SEM). Comparisons are obtained with one-way ANOVA test for normally distributed values followed by Holm-Sidak's multiple comparison test. P-values are reported in the graphs**.**

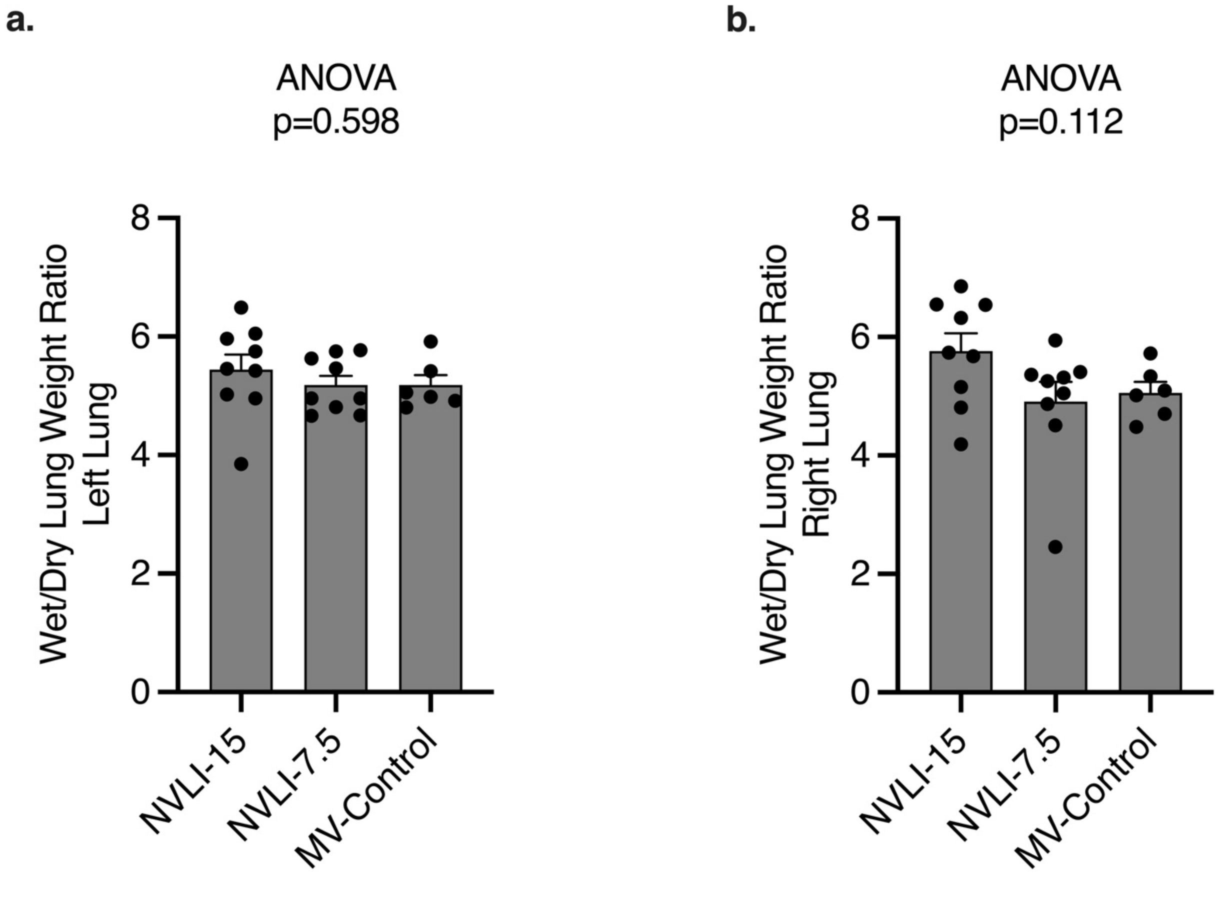

Supplementary Figure 2. Absolute value of left lung perfusion and pH trends along all study timepoints. Data in panels A-B are expressed as mean ± SEM. Comparisons are obtained by mixed-effect model for repeated measurements (results from Tukey's multiple comparison test can be found in the online supplement). P-values are reported in the graph.

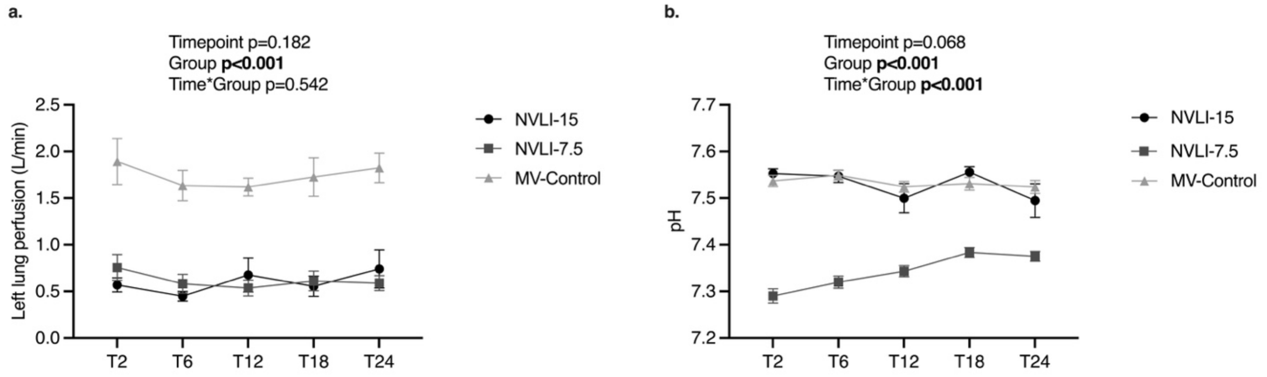

Supplement: Supplementary file 2 — Supplementary Information [file 43856_2024_449_MOESM2_ESM.pdf]
