# Supplementary material for: Pathophysiological profile of non-ventilated lung injury in healthy female pigs undergoing mechanical ventilation
Source: Commun Med (Lond). 2024 Feb 15;4:18. doi: 10.1038/s43856-024-00449-3 (PMC10869686; doi:10.1038/s43856-024-00449-3)
Supplement: Supplementary file 3 — Description of Additional Supplementary Files [file 43856_2024_449_MOESM3_ESM.pdf]

Description of Additional Supplementary Files

File Name: Supplementary Data 1
Description: This supplementary table contains all the physiological variables measured at each time point from the 3 study groups.

File Name: Supplementary Data 2
Description: This file contains the raw data used to perform all study analyses
