# Supplementary material for: Pathophysiological profile of non-ventilated lung injury in healthy female pigs undergoing mechanical ventilation
Source: Commun Med (Lond). 2024 Feb 15;4:18. doi: 10.1038/s43856-024-00449-3 (PMC10869686; doi:10.1038/s43856-024-00449-3)
Supplement: Supplementary file 6 — Reporting Summary [file 43856_2024_449_MOESM6_ESM.pdf]

nature portfolio

Corresponding author(s): Tommaso Mauri

Last updated by author(s): 2023/26/11

Reporting Summary

Nature Portfolio wishes to improve the reproducibility of the work that we publish. This form provides structure for consistency and transparency in reporting. For further information on Nature Portfolio policies, see our Editorial Policies and the Editorial Policy Checklist.

Statistics

For all statistical analyses, confirm that the following items are present in the figure legend, table legend, main text, or Methods section.

n/a	Confirmed	
☐	☒	The exact sample size (*n*) for each experimental group/condition, given as a discrete number and unit of measurement
☐	☒	A statement on whether measurements were taken from distinct samples or whether the same sample was measured repeatedly
☐	☒	The statistical test(s) used AND whether they are one- or two-sided<br>*Only common tests should be described solely by name; describe more complex techniques in the Methods section.*
☒	☐	A description of all covariates tested
☐	☒	A description of any assumptions or corrections, such as tests of normality and adjustment for multiple comparisons
☐	☒	A full description of the statistical parameters including central tendency (e.g. means) or other basic estimates (e.g. regression coefficient) AND variation (e.g. standard deviation) or associated estimates of uncertainty (e.g. confidence intervals)
☒	☐	For null hypothesis testing, the test statistic (e.g. *F*, *t*, *r*) with confidence intervals, effect sizes, degrees of freedom and *P* value noted<br>*Give P values as exact values whenever suitable.*
☒	☐	For Bayesian analysis, information on the choice of priors and Markov chain Monte Carlo settings
☒	☐	For hierarchical and complex designs, identification of the appropriate level for tests and full reporting of outcomes
☒	☐	Estimates of effect sizes (e.g. Cohen's *d*, Pearson's *r*), indicating how they were calculated

Our web collection on statistics for biologists contains articles on many of the points above.

Software and code

Policy information about availability of computer code

Data collection	Excel, Microsoft
Data analysis	GraphPad Software, San Diego, CA

For manuscripts utilizing custom algorithms or software that are central to the research but not yet described in published literature, software must be made available to editors and reviewers. We strongly encourage code deposition in a community repository (e.g. GitHub). See the Nature Portfolio guidelines for submitting code & software for further information.

Data

Policy information about availability of data

All manuscripts must include a data availability statement. This statement should provide the following information, where applicable:

- Accession codes, unique identifiers, or web links for publicly available datasets
- A description of any restrictions on data availability
- For clinical datasets or third party data, please ensure that the statement adheres to our policy

The datasets used and/or analysed during the current study are available from the corresponding author on reasonable request

Human research participants

Policy information about studies involving human research participants and Sex and Gender in Research.

Reporting on sex and gender	n/a
Population characteristics	n/a
Recruitment	n/a
Ethics oversight	n/a

Note that full information on the approval of the study protocol must also be provided in the manuscript.

Field-specific reporting

Please select the one below that is the best fit for your research. If you are not sure, read the appropriate sections before making your selection.

☒ Life sciences ☐ Behavioural & social sciences ☐ Ecological, evolutionary & environmental sciences

For a reference copy of the document with all sections, see nature.com/documents/nr-reporting-summary-flat.pdf

Life sciences study design

All studies must disclose on these points even when the disclosure is negative.

Sample size	Difference in the histological score of the left lung between the NVLI-15 vs. MV-Control group was the primary endpoint of the study. Sample size was comparable to previous animal studies on similar topic [28, 31]. We also performed a power analysis by hypothesizing, based on previous studies [31-32], values of the histological scores of 10.5±3.0 vs. 5.0±3.0: to obtain power of 0.8 with alpha 0.05. The minimum sample size resulted in 5 animals per group.
Data exclusions	No data were excluded
Replication	All experiments were repeated in different animals with no problem
Randomization	Randomized study (computer generated)
Blinding	Blinding was not possible

Reporting for specific materials, systems and methods

We require information from authors about some types of materials, experimental systems and methods used in many studies. Here, indicate whether each material, system or method listed is relevant to your study. If you are not sure if a list item applies to your research, read the appropriate section before selecting a response.

Materials & experimental systems

n/a	Involved in the study	
☒	☐	Antibodies
☒	☐	Eukaryotic cell lines
☒	☐	Palaeontology and archaeology
☐	☒	Animals and other organisms
☒	☐	Clinical data
☒	☐	Dual use research of concern

Methods

n/a	Involved in the study	
☒	☐	ChIP-seq
☒	☐	Flow cytometry
☒	☐	MRI-based neuroimaging

Animals and other research organisms

Policy information about studies involving animals; ARRIVE guidelines recommended for reporting animal research, and Sex and Gender in Research

Laboratory animals	Sus scrofa domesticus

Wild animals	n/a
Reporting on sex	female sex
Field-collected samples	not included
Ethics oversight	The study was approved by the Italian Ministry of Health, Rome, Italy (Auth. No. 246/2022-PR, Protocol No. 568EB.34 (ex 32)) and conducted according to the European Directive 2010/63/EU on the protection of animals used for scientific studies and the Italian decree 26/2014. Approval by the Institutional Committee for Animal Care was obtained before starting the experiments.

Note that full information on the approval of the study protocol must also be provided in the manuscript.
